# Chiropractic treatment approaches for spinal musculoskeletal conditions: a cross-sectional survey

**DOI:** 10.1186/s12998-014-0033-8

**Published:** 2014-10-01

**Authors:** Mattijs Clijsters, Francesco Fronzoni, Hazel Jenkins

**Affiliations:** Macquarie University Sydney, Sydney, NSW 2109 Australia; Department of Chiropractic, Macquarie University Sydney, Sydney, NSW 2109 Australia

**Keywords:** Chiropractic, Technique systems, Manipulation, Manual therapy, Musculoskeletal, Treatment, Prevalence

## Abstract

**Background:**

There are several chiropractic spinal manipulative technique systems. However, there is limited research differentiating the efficacy of these techniques. Additionally, chiropractors may also use ancillary procedures in the treatment of musculoskeletal pain, a variable that also needs to be considered when measuring the efficacy of chiropractic therapy. No data is currently available regarding the frequency of usage of chiropractic technique systems or ancillary procedures for the treatment of specific musculoskeletal conditions. Knowing which technique systems and ancillary procedures are used most frequently may help to direct future research. The aim of this research was to provide insight into which treatment approaches are used most frequently by Australian chiropractors to treat spinal musculoskeletal conditions.

**Methods:**

Cross-sectional survey design. The survey was sent online to the members of the two main Australian chiropractic associations between 30^th^ June 2013 and 7^th^ August 2013. The participants were asked to provide information on treatment choices for specific spinal musculoskeletal conditions.

**Results:**

280 respondents. Diversified manipulative technique was the first choice of treatment for most of the included conditions. Diversified was used significantly less in 4 conditions; cervical disc syndrome with radiculopathy and cervical central stenosis were more likely to be treated with Activator; flexion distraction technique was used almost as much as Diversified in the treatment of lumbar disc syndrome with radiculopathy and lumbar central stenosis. More experienced Australian chiropractors use more Activator and soft tissue therapy and less Diversified technique compared to their less experienced peers. The majority of responding chiropractors used ancillary procedures such as soft tissue techniques and exercise prescription in the treatment of spinal musculoskeletal conditions.

**Conclusion:**

This survey provides information on commonly used treatment choices to the chiropractic profession. Treatment choices changed based on the region of disorder and whether neurological symptoms were present rather than with specific diagnoses. Diversified technique was the most commonly used spinal manipulative therapy, however, ancillary procedures such as soft tissue techniques and exercise prescription were also commonly utilised. This information may help direct future studies into the efficacy of chiropractic treatment for spinal musculoskeletal disorders.

**Electronic supplementary material:**

The online version of this article (doi:10.1186/s12998-014-0033-8) contains supplementary material, which is available to authorized users.

## Background

One of the main tools chiropractors use to treat patients is the chiropractic manipulation, which can be manually applied or instrument-assisted. In the chiropractic profession there are several technique systems with regard to spinal manipulative therapy [1]. Curiously, in studies that examine the effect of spinal manipulation the technique system used is often not described, or a variety of techniques are applied in the intervention [2,3]. As different chiropractic techniques might cause distinct effects, the results of such intervention studies do not reveal information of the effectiveness of a single technique system. Furthermore, a particular system might be more or less effective depending on the musculoskeletal condition it is used for. In chiropractic research studies the targeted musculoskeletal condition is often not specified. General symptomatic areas such as neck pain are researched instead of more defined conditions such as cervical facet syndrome or cervical disc syndrome. In medicine, the condition to be treated and the exact drug are specifically described and tested. For example “the efficacy of …acyclovir…in the treatment of post-herpetic pain” [4]. By doing this they know the exact effectiveness of the drug for that specific condition. If future chiropractic studies could administer manipulations from only one chiropractic technique system targeted to a specific musculoskeletal condition, it would enhance the study’s clinical relevancy.

There are several commonly used chiropractic technique systems [1] and many different spinal musculoskeletal conditions, therefore a myriad of specific intervention studies would have to be executed to cover all clinical situations. To aid in this process our survey aims to explore which techniques graduate chiropractors most frequently use to treat common musculoskeletal conditions. Frequency of use of a certain technique system in the treatment of a particular condition is not evidence of its effectiveness. However, it indicates that further research needs to be prioritised to these techniques to produce resultant data that will be relevant to a large group of chiropractors. As chiropractors do not only use manipulation in their treatment approaches [5-12], this study will also explore the usage of ancillary treatment techniques such as soft tissue therapy and exercise prescription.

Previous published studies have already explored the frequency of usage of chiropractic technique systems in general in clinical practice [5,13,14]. Our study will explore the frequency of usage of these technique systems in particular musculoskeletal conditions. With regards to the specific conditions we surveyed there is only limited positive evidence available in the literature for manipulative treatment (in isolation or as part of the therapy) of cervicogenic headache [15], myofascial pain syndrome [10], cervical radiculopathy [16], lumbar disc syndrome [2], lumbar stenosis [17], lumbar disc herniation [18,19] and sacroiliac dysfunction [20]. However, the evidence is weak due to lack of randomised controlled trials. Most studies included in the referenced reviews did not include any specification of the used technique system for manipulation.

The purpose of this survey is to provide descriptive information to help inform researchers and chiropractors about the patterns of use of chiropractic techniques by Australian chiropractors in specific musculoskeletal conditions. In particular, this study aims to provide a starting point for future intervention studies.

## Methods

The study, an online cross-sectional survey of Australian chiropractors, was approved by the Macquarie University Human Research Ethics Committee (Medical Sciences) (reference no.: 5201300295) prior to the commencement of the study.

### Survey development

The research being undertaken has not been previously performed in the literature and as such a relevant validated survey could not be found. Therefore, the survey questions were developed for initial use in this study. A list of commonly treated musculoskeletal conditions and commonly used chiropractic modalities was created based on literature review and consultation with practicing chiropractors. This process resulted in a list of 18 common spinal musculoskeletal conditions and eight chiropractic technique systems or ancillary procedures.

The final survey included background demographic questions and questions regarding most commonly used treatment modalities. For each of the spinal musculoskeletal conditions the participants were asked to select their first, second and third most commonly used treatment modalities. Where less than three modalities were used for a particular condition, participants were instructed to leave the additional modalities blank. Participants were given the opportunity to select ‘other’ as a treatment modality and any additional techniques used could be specified at the end of the survey. The survey questions used in this research can be found in Additional file 1.

The survey was pilot-tested in the Department of Chiropractic at Macquarie University. Eight staff members (graduate chiropractors involved in education) completed the online survey and provided feedback about the content and accessibility of the survey, with subsequent minor amendments made. The final version of the online survey was structured to allow participants to complete it within a five to ten minute time period.

### Survey administration and data management

The online survey was emailed via the professional associations COCA and CAA to their members. They have approximately 1000 and 2700 members respectively [21,22]. An initial email to the participants was followed by a reminder email after three weeks. The survey was open from the 30^th^ of June 2013 until the 7^th^ of August 2013. All potential participants were notified that participation was voluntary and that confidentiality would be maintained. No identifying information was requested.

The survey was designed and administered online using the Qualtrics software of the Qualtrics Research Suite (Qualtrics, Provo, UT) [23].

Survey response rates were calculated compared to the number of chiropractors in the professional associations and the number of chiropractors within Australia. Demographic data from survey respondents was compared to national demographic data from the Chiropractic Board of Australia. Descriptive statistics were used to describe the style of practice reported and the main techniques generally used by respondents. Descriptive statistics were also used to summarise the overall frequency of individual techniques used for each musculoskeletal condition and the most commonly used techniques as first choice of treatment. Finally responses were subdivided into those from practitioners with more than ten years’ experience and those from practitioners with less than ten years’ experience. Descriptive statistics were used to describe any differences in treatment techniques between these two groups.

## Results

### Response rates

Two hundred and eighty practitioners completed the online survey, giving a response rate of 7%. However, this is likely to be an underestimation of the true response rate. It is unknown how many chiropractors are members of both professional associations, therefore, the total number of chiropractors who received the email is likely to be less than 3700. In addition, it is unknown how many members successfully received and opened the email invitation to participate in the survey. The number of total practicing registered chiropractors in Australia is 4399 [24], the available data, therefore, represented 6% of the total number of chiropractors working in Australia.

### Demographics and background data

As reported in Table 1, 58% of the respondents were under 40 years old and half of the respondents have been in practice for ten years or less. Fifty-seven percent of the participants received their education in New South Wales (NSW). Almost half of the respondents (47%) are practising in NSW, whereas only 16% are practising in Victoria. Only 3 respondents were from New Zealand.

**Table 1:** **Demographic and background data**

	**Responses (n)**	**%**
**Age of the participants (n = 263)**		
<26	24	9%
26-30	57	22%
31-35	44	17%
36-40	27	10%
41-45	26	10%
46-50	21	8%
51-55	27	10%
56-60	20	8%
61-65	8	3%
>65	9	3%
**Place of Chiropractic Education (n = 263)**		
NSW	151	57%
VIC	64	24%
WA	15	6%
New Zealand	3	1%
Other (please specify)	30	11%
**Place of Practice (n = 267)**		
NSW	122	47%
VIC	43	16%
SA	20	8%
ACT	5	2%
QLD	35	13%
TAS	4	2%
WA	23	9%
NT	0	0%
New Zealand	2	1%
Other (Please specify)	13	5%
**Years in Practice (n = 264)**		
<6	96	36%
6-10	37	14%
11-15	38	14%
16-20	24	9%
21-30	39	15%
31-40	25	9%
>40	5	2%

Key: NSW = New South Wales; VIC = Victoria; WA = Western Australia; SA = South Australia; ACT = Australian Capital Territory; QLD = Queensland; TAS = Tasmania; NT = Northern Territory.

When compared to chiropractic registrant data from the Chiropractic Board of Australia [24], demographic distribution of the survey respondents is skewed towards younger practitioners and those practising in NSW; and away from those practising in Victoria. Chiropractic registrant data reports 34% of Australian chiropractors practicing in NSW, 27% in Victoria and 50% less than 40 years old. The percentages of respondents from other states are similar to reports from the Chiropractic Board of Australia [24].

### Scope of practice and main technique used in practice

The survey also included a question on scope of practice. As seen in Table 2, 97% of respondents described their scope of practice to be based on treatment of musculoskeletal pain and/or dysfunction. Ninety-six percent of the respondents reported use of rehabilitation or exercise prescription in their treatments. Ninety-seven percent of the respondents declared they used an evidence informed approach in their daily practice.

**Table 2:** **Scope of practice**

	**Always**	**Most of the time**	**Sometimes**	**Rarely**	**Never**	**Total responses**
Wellness care	51 (20%)	74 (29%)	63 (25%)	35 (14%)	28 (11%)	251
Subluxation-based care	42 (17%)	49 (20%)	37 (15%)	33 (13%)	89 (36%)	250
Treatment of musculoskeletal dysfunction	169 (65%)	72 (29%)	11 (4%)	4 (2%)	3 (1%)	259
Treatment of musculoskeletal pain	142 (55%)	84 (33%)	25 (10%)	2 (1%)	5 (2%)	258
Evidence informed practice	112 (44%)	112 (44%)	25 (10%)	4 (2%)	3 (1%)	256
Rehabilitation or exercise prescription	92 (36%)	104 (41%)	49 (19%)	9 (4%)	2 (1%)	256
Other (Please specify)	13 (31%)	14 (33%)	8 (19%)	1 (2%)	6 (14%)	42

Chiropractors were also asked about the main technique system they used in practice. The majority of them (67%) used Diversified, followed by instrument adjusting (5%), Gonstead technique (5%) and Thompson or table assisted drop piece technique (4%). Seventeen percent of respondents reported that they used ‘other’ techniques. On analysis of their responses no clear technique systems were being repetitively used and a number of respondents had used the ‘other’ response to account for using more than one of the technique systems listed in the survey.

### Techniques used for specific musculoskeletal disorders

Table 3 summarises the overall frequency of use of each technique for the musculoskeletal conditions surveyed. Diversified technique, soft tissue therapy, instrument adjusting and exercise prescription are the most commonly used techniques throughout the cervical and thoracic spinal regions, regardless of condition. In the lumbar spine instrument adjusting is less commonly used and table assisted drop piece/Thompson technique and ‘other’ techniques become more common. Flexion distraction also demonstrates increased usage in the lumbar spine, particularly with disorders associated with neurological change including lumbar disc syndrome (with radiculopathy), lumbar lateral canal stenosis and lumbar central canal stenosis.

**Table 3:** Overall frequency of use of each technique for specific musculoskeletal conditions*

**Musculoskeletal condition**	**Overall order of use of techniques**								
	**1** ^**st**^	**2** ^**nd**^	**3** ^**rd**^	**4** ^**th**^	**5** ^**th**^	**6** ^**th**^	**7** ^**th**^	**8** ^**th**^	**9** ^**th**^
Cervical myofascial pain syndrome	STT (84%)	Div (78%)	Ex (52%)	Instr (36%)	Other (21%)	Gon (8%)	TPT (6%)	EPT (4%)	FlexDist (<1%)
Torticollis	STT (82%)	Div (76%)	Instr (43%)	Ex (38%)	Other (25%)	Gon (7%)	EPT (5%)	TPT (5%)	FlexDist (2%)
Cervical facet syndrome	Div (85%)	STT (66%)	Ex (44%)	Instr (42%)	Other (18%)	Gon (9%)	TPT (7%)	EPT (4%)	FlexDist (2%)
Cervical disc syndrome (without radiculopathy)	Div (66%)	STT (65%)	Instr (45%)	Ex (45%)	Other (25%)	TPT (8%)	FlexDist (7%)	Gon (7%)	EPT (7%)
Cervical disc syndrome (with radiculopathy)	STT (65%)	Instr (50%)	Div (46%)	Ex (45%)	Other (36%)	FlexDist (11%)	EPT (9%)	TPT (7%)	Gon (6%)
Cervical lateral stenosis	STT (67%)	Div (59%)	Ex (50%)	Instr (44%)	Other (28%)	FlexDist (6%)	TPT (6%)	Gon (5%)	EPT (5%)
Cervical central stenosis	STT (62%)	Ex (46%)	Instr (46%)	Div (38%)	Other (38%)	FlexDist (9%)	Gon (6%)	TPT (6%)	EPT (5%)
Cervical related headache	Div (85%)	STT (81%)	Ex (41%)	Instr (34%)	Other (19%)	TPT (8%)	Gon (8%)	EPT (4%)	FlexDist (1%)
Thoracic myofascial pain syndrome	Div (85%)	STT (81%)	Ex (44%)	Instr (26%)	Other (18%)	TPT (11%)	Gon (9%)	EPT (4%)	FlexDist (1%)
Thoracic facet syndrome	Div (86%)	STT (66%)	Ex (38%)	Instr (30%)	Other (17%)	TPT (16%)	Gon (13%)	EPT (4%)	FlexDist (2%)
Rib dysfunction	Div (90%)	STT (63%)	Instr (41%)	Ex (30%)	TPT (17%)	Other (15%)	Gon (7%)	EPT (4%)	FlexDist (<1%)
Lumbar myofascial pain syndrome	STT (80%)	Div (73%)	Ex (45%)	Other (23%)	Instr (23%)	TPT (20%)	Gon (12%)	EPT (5%)	FlexDist (4%)
Lumbar facet syndrome	Div (81%)	STT (61%)	Ex (42%)	TPT (27%)	Instr (25%)	Other (18%)	Gon (14%)	FlexDist (8%)	EPT (34%)
Lumbar disc syndrome (without radiculopathy)	Div (62%)	STT (53%)	Ex (47%)	TPT (29%)	Other (28%)	Instr (25%)	FlexDist (20%)	Gon (11%)	EPT (5%)
Lumbar disc syndrome (with radiculopathy)	STT (47%)	Ex (47%)	Div (43%)	Other (38%)	Instr (29%)	FlexDist (29%)	TPT (26%)	Gon (10%)	EPT (9%)
Lumbar lateral stenosis	Div (55%)	STT (55%)	Ex (45%)	Instr (28%)	TPT (28%)	Other (26%)	FlexDist (21%)	Gon (10%)	EPT (4%)
Lumbar central stenosis	STT (52%)	Ex (49%)	Div (39%)	Other (35%)	Inst (29%)	FlexDist (26%)	TPT (22%)	Gon (8%)	EPT (5%)
Sacroiliac dysfunction	Div (77%)	STT (53%)	TPT (42%)	Ex (40%)	Other (30%)	Instr (20%)	Gon (13%)	EPT (2%)	FlexDist (1%)

*Percentages add up to more than 100% as up to 3 treatment options could be selected per condition.

Key: STT = soft tissue therapy. TPT = Table assisted drop piece/Thompson technique. Instr = Instrument adjusting (Activator or similar). Ex = Exercise program/rehabilitation. Div = Diversified. Gon = Gonstead. FlexDist = Flexion distraction. EPT = Electrophysical therapy. Other = techniques not listed in survey.

Table 4 gives an overview of the techniques that were most commonly selected as the first treatment choice for each musculoskeletal disorder investigated. Diversified technique is the first choice of treatment modality for the majority of listed conditions. There were four conditions where there was a significant decrease in the use of Diversified as the first choice of treatment. Instrument adjusting was the first choice of treatment modality for cervical disc syndrome with radiculopathy and cervical central stenosis. Diversified technique was the preferred first treatment modality for lumbar disc syndrome with radiculopathy and lumbar central stenosis, however, flexion distraction was used with similar frequency. Soft tissue therapy and instrument adjusting were the most commonly chosen treatment modalities in combination with Diversified technique.

**Table 4:** **Techniques reported as first choice to treat specific musculoskeletal disorders**

**Musculoskeletal condition**	**Most commonly reported first choice**	**2** ^**nd**^ **most commonly reported first choice**	**3** ^**rd**^ **most commonly reported first choice**
Cervical myofascial pain syndrome	Div (46%)	STT (32%)	Instr (9%)
Torticollis	Div (40%)	STT (32%)	Instr (13%)
Cervical facet syndrome	Div (70%)	Instr (12%)	STT (7%)
Cervical disc syndrome (without radiculopathy)	Div (36%)	Instr (21%)	STT (18%)
Cervical disc syndrome (with radiculopathy)	Instr (26%)	STT (20%)	Div (18%)
Cervical lateral stenosis	Div (35%)	Instr (21%)	STT (18%)
Cervical central stenosis	Instr (23%)	Div (22%)	STT (20%)
Cervical related headache	Div (68%)	STT (12%)	Instr (8%)
Thoracic myofascial pain syndrome	Div (46%)	STT (31%)	Instr (7%)
Thoracic facet syndrome	Div (73%)	Gon (8%)	Instr (8%)
Rib dysfunction	Div (70%)	Instr (10%)	STT (8%)
Lumbar myofascial pain syndrome	Div (34%)	STT (32%)	Instr (8%)
Lumbar facet syndrome	Div (59%)	TPT (10%)	Instr (8%)
Lumbar disc syndrome (without radiculopathy)	Div (30%)	FlexDist (14%)	TPT (13%)
Lumbar disc syndrome (with radiculopathy)	Div (18%)	FlexDist (18%)	STT (16%)
Lumbar lateral stenosis	Div (30%)	FlexDist (13%)	STT (12%)
Lumbar central stenosis	Div (20%)	FlexDist (18%)	Instr (12%)
Sacroiliac dysfunction	Div (49%)	TPT (18%)	Gon (8%)

Key: STT = soft tissue therapy. TPT = Table assisted drop piece/Thompson technique. Instr = Instrument adjusting (Activator or similar). Gon = Gonstead. Div = Diversified. FlexDist = Flexion distraction.

**Figure 1:**
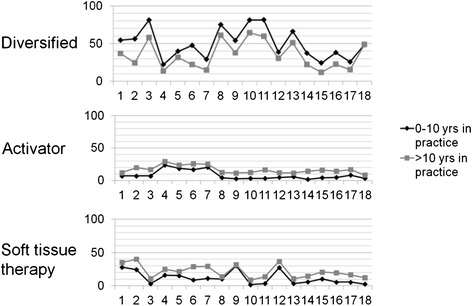
**Differences in first choice of treatment (in %) between chiropractors in practice less than 10 years versus chiropractors in practice more than 10 years.** Key: 1 Cervical myofascial pain syndrome, 2 Torticollis, 3 Cervical facet syndrome, 4 Cervical disc syndrome (without radiculopathy), 5 Cervical disc syndrome (with radiculopathy), 6 Cervical lateral stenosis, 7 Cervical central stenosis, 8 Cervical related headache, 9 Thoracic myofascial pain syndrome, 10 Thoracic facet syndrome, 11 Rib dysfunction, 12 Lumbar myofascial pain syndrome, 13 Lumbar facet syndrome, 14 Lumbar disc syndrome (without radiculopathy), 15 Lumbar disc syndrome (with radiculopathy), 16 Lumbar lateral stenosis, 17 Lumbar central stenosis, 18 Sacroiliac dysfunction.

To explore the possible role of experience in choice of technique system a comparison was made between practitioners of 10 years or less in practice (n = 133) and practitioners of more than ten years in practice (n = 131) (Figure 1). Practitioners who have been in practice ten years or less use more Diversified technique in all the conditions except for sacroiliac joint dysfunction for which Diversified was used in equal amount between the two groups. The chiropractors that have been practicing for more than a decade, use more instrument adjusting and more soft tissue therapy across all of the 18 conditions, compared to their less experienced colleagues.

## Discussion

There are many different chiropractic technique systems that have been developed. To our knowledge there is no current information available regarding which technique systems are the most effective in the management of specific musculoskeletal conditions. Developing studies to evaluate the effect of every technique system on every specific condition is not feasible at this stage. This survey describes the techniques commonly used by chiropractors in the treatment of specific spinal musculoskeletal conditions with the aim to help researchers make clinically relevant choices for future research.

### Scope of practice

The majority of respondents primarily focus their treatments on musculoskeletal conditions and apply an evidence informed approach to their clinical practice (Table 2). Therefore, the scope of practice reported by the respondents is consistent with the focus of the survey. The positive attitude of many Australian chiropractors towards evidence based practice was also found in a study from Walker et al., where 78% of the respondents agreed that the application of evidence based practice is necessary [25].

Diversified technique was reported to be the most commonly used technique system amongst Australian chiropractors. The high frequency of use of Diversified technique is in line with previous studies from Australia and overseas [4-6,13,26]. A Canadian study from 2009 found that Diversified was the main technique used in private practice, followed by Activator and Thompson technique [14]. In North America, Diversified technique is by far the most common (over 92%), followed by flexion distraction, Gonstead and Activator [5]. In 1994 a large chiropractic job analysis was done in Australia and New Zealand [13]. At that time Diversified was the most commonly used technique, followed by Activator, Gonstead, SOT, AK, Thompson and flexion distraction. In 2005, Walker et al. [26] conducted a telephone survey in Australia and New Zealand. In this study the most common technique system used by Australasian practitioners was Activator (49%), followed by Diversified (44%) and Gonstead (29%). However, additional categories of ‘manual adjustment’ and ‘manipulation’ were used in Walker’s survey that may have skewed the results.

The survey results indicate that Australian chiropractors often include exercise prescription and soft tissue therapy in their treatments but rarely use electrophysical therapies. This is in contrast to chiropractic care in North America [5,27] but similar to European studies [6,28]. French et al. [29] performed an observation and analysis study of Australian chiropractors. They found a high use of manipulative technique, soft tissue techniques and exercise prescription consistent with the results of this survey.

### Technique selection for specific musculoskeletal conditions

Manipulative therapy (Diversified technique), soft tissue techniques and exercise prescription were reported as the most commonly used treatment techniques in the management of spinal musculoskeletal disorders. Instrument adjusting (Activator or similar) was commonly used in the cervical spine, however, use decreased in the thoracic and lumbar spinal regions. Table assisted drop piece and flexion distraction techniques were more commonly used in the lumbar spine. Small changes were noted in the frequency of use of different techniques between specific musculoskeletal conditions, however, the predominant differences were region rather than condition specific.

Diversified manipulative technique is the most frequent initial treatment of choice for the majority of musculoskeletal conditions surveyed. In 16 of the listed 18 conditions, it was reported to be used as the most frequent first choice of treatment. Conditions with a neural component such as: cervical disc syndrome (with radiculopathy); cervical central stenosis; lumbar disc syndrome (with radiculopathy); and lumbar central stenosis were associated with less use of Diversified technique as the first treatment choice. In these conditions more practitioners reported the use of instrument adjusting in the cervical spine and flexion distraction in the lumbar spine. It is unknown whether the increased use of instrument adjustment and flexion distraction in these conditions may be related to safety concerns or belief of increased efficacy. Instrument adjusting and flexion distraction are viewed as lower force techniques, however, no clinical evidence exists indicating that the use of these techniques is safer than Diversified technique [30]. Further research to determine risk versus treatment benefit is important in these cases.

A higher use of instrument adjusting (Activator or similar) was reported for musculoskeletal conditions in the cervical spine compared to conditions in other spinal regions. Similar findings were reported in a British study where chiropractors reported cervical pain as the predominant reason for using Activator [31]. Our data suggests an increased use of flexion distraction in conditions such as lumbar disc syndrome with radiculopathy and lumbar central stenosis. A review by Gay et al. [32] also reported that lumbar dysfunction was the main indication for the use of flexion distraction. In light of these data, controlled studies are needed to determine if instrument adjusting is more effective or safer than other treatments for cervical conditions and if flexion distraction is more effective or safer than other treatments for lumbar conditions.

Table assisted drop piece technique was rarely used for cervical and thoracic conditions, but there was an increase in use for lumbar and sacroiliac conditions. To our knowledge, no randomised trials evaluating the effectiveness of table assisted drop piece technique are available and evaluation of this technique in the treatment of sacroiliac dysfunction may be indicated.

### Factors influencing treatment choice

Chiropractors may choose to use a specific technique system in certain conditions for several different reasons. As a result of clinical experience and therapeutic trial and error in similar situations, practitioners may have developed an understanding of what techniques work better with specific presentations. Practitioners may find one technique system easier to apply than others because of their own physical characteristics or the complexity of the technique system. In addition, they may have been guided by their education and apply technique systems to a degree which they were taught in their chiropractic course.

Practitioners might choose a certain technique system, based on their clinical experience in managing patients with a similar musculoskeletal condition. A trend was noted when chiropractic practitioners of more than ten years’ of clinical experience were compared to those of less than ten years’ experience. In general, the more experienced practitioners tended to use more instrument adjusting and soft tissue therapy, whereas, the less experienced practitioners tended to use more Diversified technique. Possible reasons may be that the more experienced chiropractors have found better results with these techniques or it may relate to the fact that these techniques are less physical demanding. Also, instrument adjusting is not taught in pre-professional courses in Australia, but can be learnt after graduation. Therefore, new graduate chiropractors may use instrument adjusting less frequently due to reduced exposure to this treatment modality.

### Implications for further research

It is hard to determine which chiropractic techniques are most effective. To do this, randomised controlled trials (RCTs) have to be executed. Unfortunately, it is very difficult to provide a placebo treatment for a manipulation. RCTs comparing the clinical effectiveness of two different technique systems on specific musculoskeletal disorders may help to inform practitioners’ treatment choices. However, reaching a conclusive musculoskeletal diagnosis in a clinical setting may limit the ability to perform this research. Subgrouping musculoskeletal disorders into those with and without neurological involvement would be more achievable in a clinical setting, and would capture the differences in preferred treatment technique found in this survey. As evidenced by our data and data from other studies [5-12], a chiropractor often uses a combination of manipulative techniques and ancillary treatment methods in the clinical setting. Although this does not provide evidence of efficacy of a single technique, RCTs investigating a combined approach would more closely mimic clinical practice.

The data from this study can be used to inform future studies and direct formulation of research questions. After analysing our data we suggest seven future research questions (see ‘Proposed future research questions for major RCTs’ list below) that might directly influence decision making in clinical practice for Australian chiropractors. These seven research questions have been formulated based on the trends we described in the above sections.

### Proposed future research questions for major RCTs

- Clinical effectiveness of Diversified technique in the management of any of our listed musculoskeletal conditions.
- Clinical effectiveness of instrument adjusting (Activator or similar) in the management of cervical disc syndrome with radiculopathy.
- Clinical effectiveness of instrument adjusting (Activator or similar) in the management of cervical central stenosis.
- Clinical effectiveness of the flexion distraction technique in the management of lumbar disc syndrome with radiculopathy.
- Clinical effectiveness of the flexion distraction technique in the management of lumbar central stenosis.
- Clinical effectiveness of table assisted drop piece technique in the management of sacroiliac joint dysfunction.
- Clinical effectiveness of soft tissue therapy and/or exercise prescription in combination with Diversified technique in the management of any of our listed conditions

### Limitations

The main limitation of this research is that of low response rate. Surveys were distributed through emails from the two main Australian chiropractic associations and it is impossible to know how many chiropractors actually received and read the emails. Therefore, true response rate, and assessment of potential non-response bias, cannot be determined. Non-response bias is of concern if only subjects interested in the subject complete the survey. The results of this survey were compared to demographic data from the chiropractic registration board and previous research to try and establish how reflective the respondents of this survey were to the chiropractic population as a whole. Demographic data was similar to survey respondents except for an increase in the number of respondents working in New South Wales with a decrease in those working in Victoria and an increase in the number of respondents from a younger age group. Scope of practice among survey respondents was heavily skewed to those treating muscular pain and dysfunction, possibly indicating respondant bias. However, previous research conducted by French et al. [29] also indicated that Australian chiropractic practice primarily focuses on the treatment of musculoskeletal pain. Therefore, this result may be reflective of the chiropractic population as a whole. There was also a high proportion of respondents who used Diversified as their primary therapeutic technique as opposed to other chiropractic techniques. However, similar trends are noted in previous studies done in Australia [13,29] and the United States [5], indicating that our sample population responded fairly consistently with other, larger scaled, studies. Although we do have some similarities between the survey responses and previously published data we cannot eliminate the possiblity of non-response bias skewing the results of this survey. Therefore, the results of this survey should be interpreted with caution as they may not be reflective of the Australian chiropractic population as a whole.

Epidemiological data was to be used to help formulate the list of musculoskeletal conditions included in the survey. However, data regarding the prevalence of specific musculoskeletal conditions presenting to chiropractic practices is lacking. There is some data available regarding presenting symptomatic regions [7,26,33], but not related to specific musculoskeletal diagnoses. Therefore, selection of musculoskeletal conditions based on specific epidemiological data was not possible.

The survey instrument was not validated, however, it was based on questionnaires used in similar studies that focused on technique systems in general [5,13,14]. These questionnaires were reformed to suit our condition-specific questions. In addition, the survey was not exhaustive, with only five chiropractic technique systems included. Although the option was provided to select and specify any other technique system, the setup of the question may have influenced respondents to select one of the five listed technique systems. These five technique systems were chosen as previous research had shown them to be the main techniques used in Australia [13]. Reviewing comments from practitioners who specified “other techniques” in the survey failed to demonstrate any consistent trends in additional technique systems used.

Recall bias may also be a concern in this survey. Practitioners may over- or under-estimate the degree that they use certain techniques for specific conditions. Although we cannot rule out recall bias we feel that the general nature of the questions asked limit this as a particular concern. The survey questions asked for preferred first, second and third treatment techniques rather than the frequency of usage of those techniques to reduce the effect of recall bias.

Lastly, it may be possible that the musculoskeletal conditions listed in the survey were interpreted differently by different respondents. The aim of the survey was not to test diagnostic abilities in the practitioners, but rather to gain information about which chiropractic technique they would use to treat a specific textbook condition. Gradations in severity of the conditions were not provided, nor were many other variables that may change decision making.

## Conclusion

This survey provides information on commonly used treatment choices to the chiropractic profession. Treatment choices changed based on the region of disorder and whether neurological symptoms were present rather than with specific diagnoses. Diversified technique was the most commonly used manipulative therapy, however, ancillary procedures such as soft tissue therapy and exercise prescription were also commonly utilised. This information may help direct future studies into the efficacy of chiropractic treatment for spinal musculoskeletal disorders.

## Additional file

Additional file 1:
**Survey Questions: copy of the survey questions used in the research.**
