# Supplementary material for: Chiropractic treatment approaches for spinal musculoskeletal conditions: a cross-sectional survey
Source: Chiropr Man Therap. 2014 Oct 1;22:33. doi: 10.1186/s12998-014-0033-8 (PMC4193988; doi:10.1186/s12998-014-0033-8)
Supplement: Additional file 1: — Survey Questions: copy of the survey questions used in the research. [file 12998_2014_33_MOESM1_ESM.pdf]

Additional File 1: Copy of the survey questions

What is your age?

26-30
31-35
36-40
41-45
46-50
51-55
56-60
61-65
>65

What school/university/college did you complete your chiropractic education at?
NSW
VIC
WA
New Zealand
Other (please specify) ____________________

How many years have you been in practice?

6-10
11-15
16-20
21-30
31-40
>40

In which state do you practice? (you may select more than one response if appropriate)

- NSW
- VIC
- SA
- ACT
- QLD
- TAS
- WA
- NT
- New Zealand
- Other (Please specify) ______________________

How often do you apply the following in your practice?

	Always	Most of the Time	Sometimes	Rarely	Never
Wellness care					
Subluxation-based care					
Treatment of musculoskeletal dysfunction					
Treatment of musculoskeletal pain					
Evidence informed practice					
Rehabilitation or exercise prescription					
Other (Please specify)					

Which technique system do you mainly use in your practice?

Diversified
Gonstead
Thompson/TPT
Instrument (Activator)
Other (Please specify) ____________________

In the following questions: for each of the musculoskeletal conditions listed please select your most commonly used treatment modalities from the drop-boxes provided. If you have more than one, please select them in order of preference. If you have only one/two preferences you can leave the other box(es) blank.

Cervical myofascial pain syndrome

	Diversified	Gonstead	Thompson/TPT	Flexion Distraction	Instrument (Activator or similar)	Soft tissue therapy	Electophysical therapy	Exercise prescription	Other
1st most commonly used treatment modality									
2nd most commonly used treatment modality									
3rd most commonly used treatment modality									

Torticollis

	Diversified	Gonstead	Thompson/TPT	Flexion Distraction	Instrument (Activator or similar)	Soft tissue therapy	Electophysical therapy	Exercise prescription	Other
1st most commonly used treatment modality									
2nd most commonly used treatment modality									
3rd most commonly used treatment modality									

Cervical facet syndrome

	Diversified	Gonstead	Thompson/TPT	Flexion Distraction	Instrument (Activator or similar)	Soft tissue therapy	Electophysical therapy	Exercise prescription	Other
1st most commonly used treatment modality									
2nd most commonly used treatment modality									
3rd most commonly used treatment modality									

Cervical disc syndrome (with radiculopathy)

	Diversified	Gonstead	Thompson/TPT	Flexion Distraction	Instrument (Activator or similar)	Soft tissue therapy	Electophysical therapy	Exercise prescription	Other
1st most commonly used treatment modality									
2nd most commonly used treatment modality									
3rd most commonly used treatment modality									

Cervical disc syndrome (without radiculopathy)

	Diversified	Gonstead	Thompson/TPT	Flexion Distraction	Instrument (Activator or similar)	Soft tissue therapy	Electophysical therapy	Exercise prescription	Other
1st most commonly used treatment modality									
2nd most commonly used treatment modality									
3rd most commonly used treatment modality									

Cervical lateral stenosis

	Diversified	Gonstead	Thompson/TPT	Flexion Distraction	Instrument (Activator or similar)	Soft tissue therapy	Electophysical therapy	Exercise prescription	Other
1st most commonly used treatment modality									
2nd most commonly used treatment modality									
3rd most commonly used treatment modality									

Cervical central stenosis

	Diversified	Gonstead	Thompson/TPT	Flexion Distraction	Instrument (Activator or similar)	Soft tissue therapy	Electophysical therapy	Exercise prescription	Other
1st most commonly used treatment modality									
2nd most commonly used treatment modality									
3rd most commonly used treatment modality									

Cervical related headache

	Diversified	Gonstead	Thompson/TPT	Flexion Distraction	Instrument (Activator or similar)	Soft tissue therapy	Electophysical therapy	Exercise prescription	Other
1st most commonly used treatment modality									
2nd most commonly used treatment modality									
3rd most commonly used treatment modality									

Thoracic myofascial pain syndrome

	Diversified	Gonstead	Thompson/TPT	Flexion Distraction	Instrument (Activator or similar)	Soft tissue therapy	Electophysical therapy	Exercise prescription	Other
1st most commonly used treatment modality									
2nd most commonly used treatment modality									
3rd most commonly used treatment modality									

Thoracic facet syndrome

	Diversified	Gonstead	Thompson/TPT	Flexion Distraction	Instrument (Activator or similar)	Soft tissue therapy	Electophysical therapy	Exercise prescription	Other
1st most commonly used treatment modality									
2nd most commonly used treatment modality									
3rd most commonly used treatment modality									

Rib dysfunction

	Diversified	Gonstead	Thompson/TPT	Flexion Distraction	Instrument (Activator or similar)	Soft tissue therapy	Electophysical therapy	Exercise prescription	Other
1st most commonly used treatment modality									
2nd most commonly used treatment modality									
3rd most commonly used treatment modality									

Lumbar myofascial pain syndrome

	Diversified	Gonstead	Thompson/TPT	Flexion Distraction	Instrument (Activator or similar)	Soft tissue therapy	Electophysical therapy	Exercise prescription	Other
1st most commonly used treatment modality									
2nd most commonly used treatment modality									
3rd most commonly used treatment modality									

Lumbar facet syndrome

	Diversified	Gonstead	Thompson/TPT	Flexion Distraction	Instrument (Activator or similar)	Soft tissue therapy	Electophysical therapy	Exercise prescription	Other
1st most commonly used treatment modality									
2nd most commonly used treatment modality									
3rd most commonly used treatment modality									

Lumbar disc syndrome (without radiculopathy)

	Diversified	Gonstead	Thompson/TPT	Flexion Distraction	Instrument (Activator or similar)	Soft tissue therapy	Electophysical therapy	Exercise prescription	Other
1st most commonly used treatment modality									
2nd most commonly used treatment modality									
3rd most commonly used treatment modality									

Lumbar disc syndrome (with radiculopathy)

	Diversified	Gonstead	Thompson/TPT	Flexion Distraction	Instrument (Activator or similar)	Soft tissue therapy	Electrophysical therapy	Exercise prescription	Other
1st most commonly used treatment modality									
2nd most commonly used treatment modality									
3rd most commonly used treatment modality									

Lumbar lateral stenosis

	Diversified	Gonstead	Thompson/TPT	Flexion Distraction	Instrument (Activator or similar)	Soft tissue therapy	Electophysical therapy	Exercise prescription	Other
1st most commonly used treatment modality									
2nd most commonly used treatment modality									
3rd most commonly used treatment modality									

Lumbar central stenosis

	Diversified	Gonstead	Thompson/TPT	Flexion Distraction	Instrument (Activator or similar)	Soft tissue therapy	Electophysical therapy	Exercise prescription	Other
1st most commonly used treatment modality									
2nd most commonly used treatment modality									
3rd most commonly used treatment modality									

Sacroiliac joint dysfunction

	Diversified	Gonstead	Thompson/TPT	Flexion Distraction	Instrument (Activator or similar)	Soft tissue therapy	Electophysical therapy	Exercise prescription	Other
1st most commonly used treatment modality									
2nd most commonly used treatment modality									
3rd most commonly used treatment modality									

If you selected the response ‘other’ as a treatment modality in any of the above questions, please use the space below to describe the treatment modality that you would use.
